# Supplementary material for: Leveraging generative AI to prioritize drug repurposing candidates for Alzheimer’s disease with real-world clinical validation
Source: NPJ Digit Med. 2024 Feb 26;7:46. doi: 10.1038/s41746-024-01038-3 (PMC10897392; doi:10.1038/s41746-024-01038-3)
Supplement: Supplementary file 1 — Supplementary Material [file 41746_2024_1038_MOESM1_ESM.pdf]

Supplementary

Leveraging Generative AI to Prioritize Drug Repurposing Candidates for Alzheimer's Disease with Real-World Clinical Validation

Authors:
Chao Yan[1], Monika E. Grabowska[1], Alyson L. Dickson[2], Bingshan Li[3,4], Zhexing Wen[5,6], Dan M. Roden[2], C. Michael Stein[2], Peter J. Embí[1,2], Josh F. Peterson[1,2], QiPing Feng[2], Bradley A. Malin[1,7,8], Wei-Qi Wei[1,7]

Affiliations:
[1]Department of Biomedical Informatics, Vanderbilt University Medical Center, Nashville, TN
[2]Department of Medicine, Vanderbilt University Medical Center, Nashville, TN
[3]Department of Molecular Physiology and Biophysics, Vanderbilt University, Nashville, TN
[4]Vanderbilt Genetics Institute, Vanderbilt University Medical Center, Nashville, TN
[5]Department of Psychiatry and Behavioral Sciences, Emory University School of Medicine, Atlanta, GA
[6]Department of Cell Biology, Emory University School of Medicine, Atlanta, GA
[7]Department of Computer Science, Vanderbilt University, Nashville, TN
[8]Department of Biostatistics, Vanderbilt University Medical Center, Nashville, TN

These authors contributed equally to this research: Chao Yan and Monika E. Grabowska
These authors are senior authors of this research: Wei-Qi Wei and Bradley A. Malin

Corresponding author:
Wei-Qi Wei, MD, PhD
Associate Professor
Department of Biomedical Informatics
Vanderbilt University Medical Center
Email: wei-qi.wei@vumc.org
Address: Suite 1500, 2525 West End Ave, Nashville, TN, USA, 37203

Supplementary Table 1. The full list of drug repurposing candidates generated by ChatGPT (N=59). Drug frequencies observed across ten independent queries are indicated. Exemplary references are provided by the authors to verify the candidate's demonstrated potential for Alzheimer's disease (AD).

Drug candidate	Original purpose generated by ChatGPT	Frequency	Examples of references
Simvastatin	Hypercholesterolemia	10	1,2
Metformin	Type 2 diabetes	10	3,4
Losartan	Hypertension	10	5,6
Pioglitazone	Type 2 Diabetes	10	7,8
Candesartan	Hypertension	9	9.10
Rapamycin	Organ transplant rejection	9	11,12
Bexarotene	Cutaneous T-cell lymphoma	8	13,14
Lithium	Bipolar disorder	7	15,16
Minocycline	Bacterial infections	7	17,18
Nilotinib	Chronic Myeloid Leukemia	7	19,20
Montelukast	Asthma and allergies	6	21,22
Saracatinib	Cancer (solid tumors)	6	23,24
Fluoxetine	Depression	6	25,26
Carvedilol	Congestive heart failure, hypertension	6	27,28
Riluzole	Amyotrophic lateral sclerosis	5	29,30
Citalopram	Depression	5	31,32
Doxycycline	Bacterial infections	5	33,34
Atorvastatin	Hypercholesterolemia	4	35,36
Masitinib	Cancer (gastrointestinal stromal tumors)	4	37,38
Nilvadipine	Hypertension	3	39,40
Mebendazole	Parasitic Infections	3	41
Levetiracetam	Epilepsy	3	42,43
Ibudilast	Asthma	3	44,45
Methylene blue	Malaria	3	46,47

Latrepirdine	Allergies	3	48,49
Valproate	Epilepsy	2	50,51
Trazodone	Depression	2	52,53
Nortriptyline	Depression	2	54
Methotrexate	Rheumatoid Arthritis	2	55,56
J147	Neurological dysfunction	2	57,58
Rosiglitazone	Type 2 Diabetes	2	59,60
Telmisartan	Hypertension	2	61,62
Exenatide	Type 2 diabetes	2	63,64
Etanercept	Rheumatoid Arthritis	2	65,66
Dextromethorphan	Cough	2	67,68
Valproic acid	Epilepsy	1	69,70
Valsartan	Hypertension	1	71,72
Sodium valproate	Epilepsy	1	50
Tolcapone	Parkinson's disease	1	73
Rivaroxaban	Deep vein thrombosis and pulmonary embolism	1	74
Nebivolol	Hypertension	1	75
Sildenafil	Erectile dysfunction	1	76
Acamprosate	Alcohol dependence	1	77
Naproxen	Inflammation	1	78
Colchicine	Gout	1	79
Amiloride	Hypertension	1	80
Amitriptyline	Depression and Neuropathic Pain	1	81
Azeliragon	Type 2 Diabetes*	1	82
Baclofen	Muscle Spasticity	1	83
Cannabidiol	Epilepsy	1	84
Cimetidine	Peptic Ulcers	1	85
Clemastine	Allergy, Hay fever	1	86

Dexamethasone	Inflammation	1	87
Naltrexone	Opioid and alcohol dependence	1	88
Exendin-4	Type 2 diabetes	1	89
Ganaxolone	Epilepsy	1	90
Hydroxychloroquine	Malaria, Rheumatoid Arthritis, Lupus	1	91
Ibuprofen	Pain and Inflammation	1	92
Intranasal insulin	Diabetes	1	93

*Azeliragon was first tested for use in AD by Pfizer, but has been proposed for treating a variety of diseases, including type 2 diabetes, glioblastoma, and cancer.

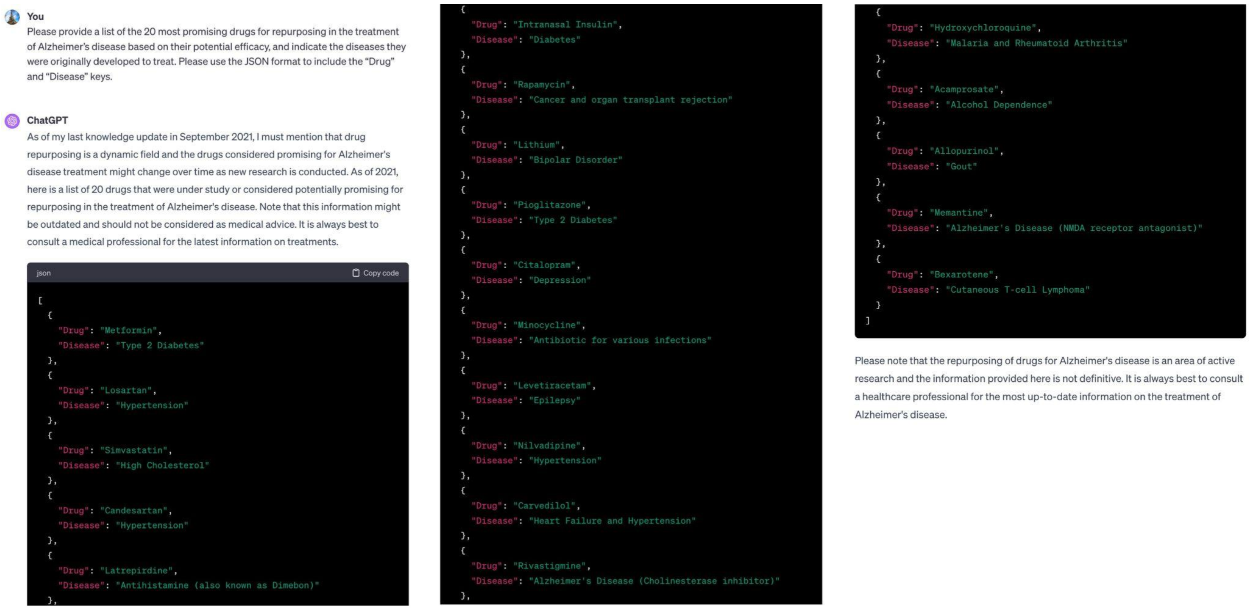

Supplementary Figure 1. An example of ChatGPT prompt and response (screenshots) for generating drug repurposing candidates.

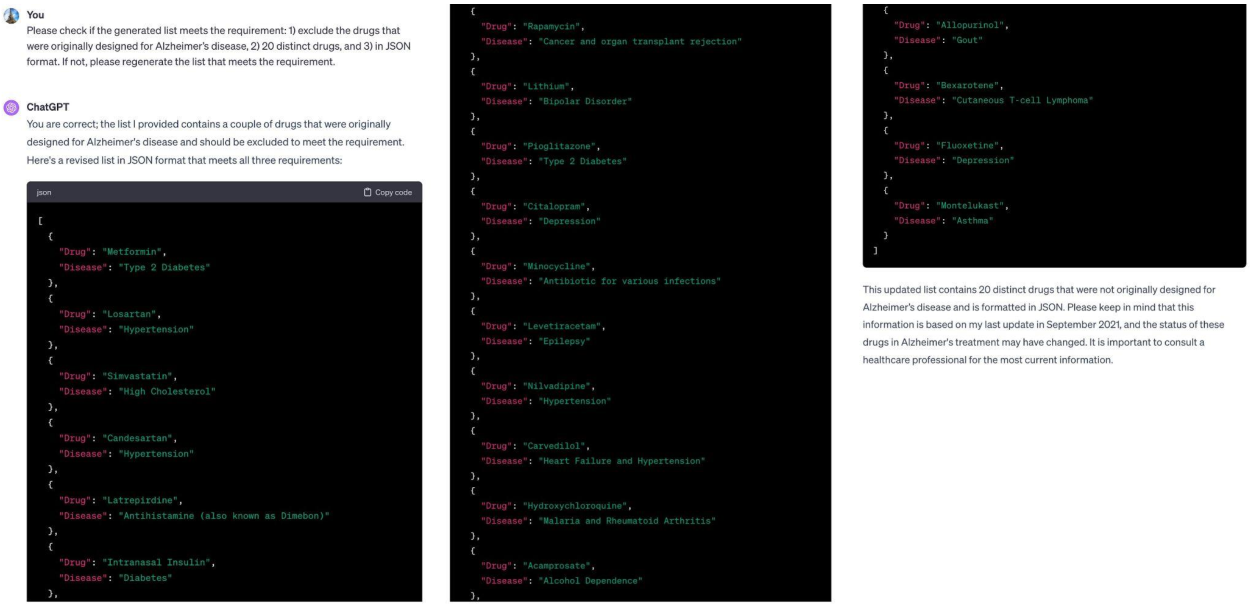

Supplementary Figure 2. An example of ChatGPT prompt and response (screenshots) for self-correction.

Supplementary Table 2. International Classification of Diseases (ICD-9-CM and ICD-10-CM) codes used to define drug-specific comorbidities based on primary clinical indication.

Drug	Clinical indication	Diagnosis codes
Lithium	Bipolar or depressive disorder	**ICD-9-CM:** 296.80, 311<br>**ICD-10-CM:** F31.9, F32.9
Metformin, Pioglitazone	Type 2 diabetes	**ICD-9-CM:** 250.00<br>**ICD-10-CM:** E11.9
Simvastatin	Hyperlipidemia	**ICD-9-CM:** 272.0, 272.2, 272.4, 413.9, 414.00, 786.50<br>**ICD-10-CM:** E78.2, E78.5, I20.8, I20.9, I25.10, R07.9
Candesartan, Losartan	Hypertension	**ICD-9-CM:** 401.1, 401.9<br>**ICD-10-CM:** I10
Rapamycin	N/A	N/A
Nilotinib	N/A	N/A
Minocycline	N/A	N/A
Bexarotene	N/A	N/A

Supplementary Table 3. Alzheimer's disease (AD) event counts stratified by drug exposure status for the ten drug repurposing candidates most frequently recommended by ChatGPT. In accordance with the *All of Us* Data and Statistics Dissemination Policy, participant counts between 1 and 20 are not reported directly.

	**VUMC** (N)				**All of Us** (N)			
	**Exposed**		**Unexposed**		**Exposed**		**Unexposed**	
**Drug**	**AD**	**No AD**	**AD**	**No AD**	**AD**	**No AD**	**AD**	**No AD**
Metformin	145	23,730	217	47,533	≤20	5,209	28	10,422
Losartan	93	17,868	255	35,667	≤20	3,591	≤20	7,186
Minocycline	5	1,516	29	3,013	0	396	≤20	790
Simvastatin	260	32,705	458	65,472	≤20	7,081	48	14,142
Pioglitazone	25	3,925	67	7,833	≤20	1,306	≤20	2,604
Candesartan	16	1,398	26	2,802	0	144	0	288
Rapamycin	1	294	4	586	0	60	0	120
Lithium	9	778	16	1,558	0	182	≤20	362
Bexarotene	0	108	3	213	0	≤20	0	≤20
Nilotinib	0	33	1	65	0	≤20	0	≤20

Supplementary Table 4. Standardized mean differences for covariates after propensity score matching.

	VUMC				All of Us			
**Drug**	**Sex**	**Race**	**EHR length after 65**	**Comorbidity**	**Sex**	**Race**	**EHR length after 65**	**Comorbidity**
Metformin	0.0161	-0.0342	0.5734	1.0143	-0.0261	-0.0472	0.4973	1.2600
Losartan	0.0030	-0.0014	0.0002	0.0002	-0.0102	0.0031	0.0002	0.0000
Minocycline	0	0	0	N/A	0	0	0	N/A
Simvastatin	-0.0125	-0.2056	0.2072	0.1983	-0.0033	-0.0170	0.0357	0.0000
Pioglitazone	0.0000	0.0023	0.0000	0.0000	-0.0287	0.0040	0.0019	-0.0016
Candesartan	0	0	0	0	-0.0242	-0.0004	0.0078	0.0000
Rapamycin	0	0	0	N/A	0.0170	0.0010	-0.0032	N/A
Lithium	-0.0026	-0.0062	0.0013	0.0000	0	0	0	0
Bexarotene	0	0	0	N/A	0	0	0	N/A
Nilotinib	0	0	0	N/A	0	0	0	N/A

References

1. Serrano-Pozo, A. *et al.* Effects of simvastatin on cholesterol metabolism and Alzheimer disease biomarkers. *Alzheimer Dis. Assoc. Disord.* 24, 220–226 (2010).
2. Langness, V. F. *et al.* Cholesterol-lowering drugs reduce APP processing to Aβ by inducing APP dimerization. *Mol. Biol. Cell* 32, 247–259 (2021).
3. Liao, W. *et al.* Deciphering the roles of metformin in Alzheimer's disease: A snapshot. *Front. Pharmacol.* 12, 728315 (2021).
4. Weinstein, G. *et al.* Association of metformin, sulfonylurea and insulin use with brain structure and function and risk of dementia and Alzheimer's disease: Pooled analysis from 5 cohorts. *PLoS One* 14, e0212293 (2019).
5. Royea, J., Zhang, L., Tong, X.-K. & Hamel, E. Angiotensin IV receptors mediate the cognitive and cerebrovascular benefits of losartan in a mouse model of Alzheimer's disease. *J. Neurosci.* 37, 5562–5573 (2017).
6. Kehoe, P. G. *et al.* Safety and efficacy of losartan for the reduction of brain atrophy in clinically diagnosed Alzheimer's disease (the RADAR trial): a double-blind, randomised, placebo-controlled, phase 2 trial. *Lancet Neurol.* 20, 895–906 (2021).
7. Miller, B. W., Willett, K. C. & Desilets, A. R. Rosiglitazone and pioglitazone for the treatment of Alzheimer's disease. *Ann. Pharmacother.* 45, 1416–1424 (2011).
8. Galimberti, D. & Scarpini, E. Pioglitazone for the treatment of Alzheimer's disease. *Expert Opin. Investig. Drugs* 26, 97–101 (2017).
9. Elkahloun, A. G., Hafko, R. & Saavedra, J. M. An integrative genome-wide transcriptome reveals that candesartan is neuroprotective and a candidate therapeutic for Alzheimer's disease. *Alzheimers. Res. Ther.* 8, 5 (2016).
10. Torika, N., Asraf, K., Apte, R. N. & Fleisher-Berkovich, S. Candesartan ameliorates brain inflammation associated with Alzheimer's disease. *CNS Neurosci. Ther.* 24, 231–242 (2018).
11. Kaeberlein, M. & Galvan, V. Rapamycin and Alzheimer's disease: Time for a clinical trial? *Sci. Transl. Med.* 11, eaar4289 (2019).
12. Cai, Z. & Yan, L.-J. Rapamycin, autophagy, and Alzheimer's disease. *J. Biochem. Pharmacol. Res.* 1, 84–90 (2013).
13. Tousi, B. The emerging role of bexarotene in the treatment of Alzheimer's disease: current evidence. *Neuropsychiatr. Dis. Treat.* 11, 311–315 (2015).
14. Cummings, J. L. *et al.* Double-blind, placebo-controlled, proof-of-concept trial of bexarotene Xin moderate Alzheimer's disease. *Alzheimers. Res. Ther.* 8, 4 (2016).
15. Matsunaga, S. *et al.* Lithium as a treatment for Alzheimer's disease: A systematic review and meta-analysis. *J. Alzheimers. Dis.* 48, 403–410 (2015).
16. Forlenza, O. V., De-Paula, V. J. R. & Diniz, B. S. O. Neuroprotective effects of lithium: implications for the treatment of Alzheimer's disease and related neurodegenerative disorders. *ACS Chem. Neurosci.* 5, 443–450 (2014).

17. Choi, Y. *et al.* Minocycline attenuates neuronal cell death and improves cognitive impairment in Alzheimer's disease models. *Neuropsychopharmacology* 32, 2393–2404 (2007).
18. Budni, J. *et al.* The anti-inflammatory role of minocycline in Alzheimer´s disease. *Curr. Alzheimer Res.* 13, 1319–1329 (2016).
19. Turner, R. S. *et al.* Nilotinib effects on safety, tolerability, and biomarkers in Alzheimer's disease. *Ann. Neurol.* 88, 183–194 (2020).
20. Lonskaya, I., Hebron, M. L., Selby, S. T., Turner, R. S. & Moussa, C. E.-H. Nilotinib and bosutinib modulate pre-plaque alterations of blood immune markers and neuro-inflammation in Alzheimer's disease models. *Neuroscience* 304, 316–327 (2015).
21. Zhang, C. T. *et al.* Montelukast ameliorates streptozotocin-induced cognitive impairment and neurotoxicity in mice. *Neurotoxicology* 57, 214–222 (2016).
22. Lai, J. *et al.* Montelukast targeting the cysteinyl leukotriene receptor 1 ameliorates Aβ1-42-induced memory impairment and neuroinflammatory and apoptotic responses in mice. *Neuropharmacology* 79, 707–714 (2014).
23. Nygaard, H. B. *et al.* A phase Ib multiple ascending dose study of the safety, tolerability, and central nervous system availability of AZD0530 (saracatinib) in Alzheimer's disease. *Alzheimers. Res. Ther.* 7, 35 (2015).
24. Kaufman, A. C. *et al.* Fyn inhibition rescues established memory and synapse loss in Alzheimer mice: Fyn Inhibition by AZD0530. *Ann. Neurol.* 77, 953–971 (2015).
25. Ma, J. *et al.* Fluoxetine attenuates the impairment of spatial learning ability and prevents neuron loss in middle-aged APPswe/PSEN1dE9 double transgenic Alzheimer's disease mice. *Oncotarget* 8, 27676–27692 (2017).
26. Zhou, C.-N. *et al.* Fluoxetine delays the cognitive function decline and synaptic changes in a transgenic mouse model of early Alzheimer's disease. *J. Comp. Neurol.* 527, 1378–1387 (2019).
27. Wang, J. *et al.* Carvedilol as a potential novel agent for the treatment of Alzheimer's disease. *Neurobiol. Aging* 32, 2321.e1–12 (2011).
28. Liu, J. & Wang, M. Carvedilol protection against endogenous Aβ-induced neurotoxicity in N2a cells. *Cell Stress Chaperones* 23, 695–702 (2018).
29. Vallée, A., Vallée, J.-N., Guillevin, R. & Lecarpentier, Y. Riluzole: a therapeutic strategy in Alzheimer's disease by targeting the WNT/β-catenin pathway. *Aging (Albany NY)* 12, 3095–3113 (2020).
30. Pereira, A. C. *et al.* Age and Alzheimer's disease gene expression profiles reversed by the glutamate modulator riluzole. *Mol. Psychiatry* 22, 296–305 (2017).
31. Porsteinsson, A. P. *et al.* Effect of citalopram on agitation in Alzheimer disease: the CitAD randomized clinical trial: The CitAD randomized clinical trial. *JAMA* 311, 682–691 (2014).
32. Leonpacher, A. K. *et al.* Effects of citalopram on neuropsychiatric symptoms in Alzheimer's dementia: Evidence from the CitAD study. *Am. J. Psychiatry* 173, 473–480 (2016).
33. Balducci, C. & Forloni, G. Doxycycline for Alzheimer's disease: Fighting β-amyloid oligomers and neuroinflammation. *Front. Pharmacol.* 10, 738 (2019).

34. Molloy, D. W., Standish, T. I., Zhou, Q., Guyatt, G. & DARAD Study Group. A multicenter, blinded, randomized, factorial controlled trial of doxycycline and rifampin for treatment of Alzheimer's disease: the DARAD trial: Doxycycline and rifampin for Alzheimer's. *Int. J. Geriatr. Psychiatry* 28, 463–470 (2013).
35. Sparks, D. L. *et al.* Atorvastatin for the treatment of mild to moderate Alzheimer disease: preliminary results: Preliminary results. *Arch. Neurol.* 62, 753–757 (2005).
36. Feldman, H. H. *et al.* Randomized controlled trial of atorvastatin in mild to moderate Alzheimer disease: LEADe. *Neurology* 74, 956–964 (2010).
37. Folch, J. *et al.* Masitinib for the treatment of mild to moderate Alzheimer's disease. *Expert Rev. Neurother.* 15, 587–596 (2015).
38. Piette, F. *et al.* Masitinib as an adjunct therapy for mild-to-moderate Alzheimer's disease: a randomised, placebo-controlled phase 2 trial. *Alzheimers. Res. Ther.* 3, 16 (2011).
39. de Jong, D. L. K. *et al.* Effects of nilvadipine on cerebral blood flow in patients with Alzheimer disease: A randomized trial. *Hypertension* 74, 413–420 (2019).
40. Kennelly, S. P. *et al.* Demonstration of safety in Alzheimer's patients for intervention with an anti-hypertensive drug Nilvadipine: results from a 6-week open label study. *Int. J. Geriatr. Psychiatry* 26, 1038–1045 (2011).
41. RePORTER. *Nih.gov* https://reporter.nih.gov/search/I615LI0fwkScy8VY5yYpcA/project-details/10185192.
42. Sanchez, P. E. *et al.* Levetiracetam suppresses neuronal network dysfunction and reverses synaptic and cognitive deficits in an Alzheimer's disease model. *Proc. Natl. Acad. Sci. U. S. A.* 109, E2895-903 (2012).
43. Musaeus, C. S., Shafi, M. M., Santarnecchi, E., Herman, S. T. & Press, D. Z. Levetiracetam alters oscillatory connectivity in Alzheimer's disease. *J. Alzheimers. Dis.* 58, 1065–1076 (2017).
44. Wang, H. *et al.* Pretreatment with antiasthmatic drug ibudilast ameliorates Aβ 1-42-induced memory impairment and neurotoxicity in mice. *Pharmacol. Biochem. Behav.* 124, 373–379 (2014).
45. Oliveros, G. *et al.* Multi-scale predictive modeling discovers Ibudilast as a polypharmacological agent to improve hippocampal-dependent spatial learning and memory and mitigate plaque and tangle pathology in a transgenic rat model of Alzheimer's disease. *bioRxiv* (2021) doi:10.1101/2021.04.06.438662.
46. Oz, M., Lorke, D. E. & Petroianu, G. A. Methylene blue and Alzheimer's disease. *Biochem. Pharmacol.* 78, 927–932 (2009).
47. Paban, V. *et al.* Therapeutic and preventive effects of methylene blue on Alzheimer's disease pathology in a transgenic mouse model. *Neuropharmacology* 76 Pt A, 68–79 (2014).
48. Chau, S., Herrmann, N., Ruthirakuhan, M. T., Chen, J. J. & Lanctôt, K. L. Latrepirdine for Alzheimer's disease. *Cochrane Database Syst. Rev.* CD009524 (2015) doi:10.1002/14651858.CD009524.pub2.

49. Bharadwaj, P. R. *et al.* Latrepirdine: molecular mechanisms underlying potential therapeutic roles in Alzheimer's and other neurodegenerative diseases. *Transl. Psychiatry* 3, e332–e332 (2013).
50. Yao, Z.-G. *et al.* Valproate improves memory deficits in an Alzheimer's disease mouse model: investigation of possible mechanisms of action. *Cell. Mol. Neurobiol.* 34, 805–812 (2014).
51. Hu, J.-P. *et al.* Valproate reduces tau phosphorylation via cyclin-dependent kinase 5 and glycogen synthase kinase 3 signaling pathways. *Brain Res. Bull.* 85, 194–200 (2011).
52. La, A. L. *et al.* Long-term trazodone use and cognition: A potential therapeutic role for slow-wave sleep enhancers. *J. Alzheimers. Dis.* 67, 911–921 (2019).
53. Ashford, J. W. Treatment of Alzheimer's disease: Trazodone, sleep, serotonin, norepinephrine, and future directions. *J. Alzheimers. Dis.* 67, 923–930 (2019).
54. Bartlomé, P., King, K. S., Matsuo, F. & Wood, J. S. Cognitive decline with nortriptyline use in a patient with dementia of the Alzheimer's type. *West. J. Med.* 156, 75–77 (1992).
55. Koźmiński, P., Halik, P. K., Chesori, R. & Gniazdowska, E. Overview of dual-acting drug methotrexate in different neurological diseases, autoimmune pathologies and cancers. *Int. J. Mol. Sci.* 21, 3483 (2020).
56. Newby, D. *et al.* Methotrexate and relative risk of dementia amongst patients with rheumatoid arthritis: a multi-national multi-database case-control study. *Alzheimers. Res. Ther.* 12, 38 (2020).
57. Prior, M., Dargusch, R., Ehren, J. L., Chiruta, C. & Schubert, D. The neurotrophic compound J147 reverses cognitive impairment in aged Alzheimer's disease mice. *Alzheimers. Res. Ther.* 5, 25 (2013).
58. Prior, M. *et al.* Selecting for neurogenic potential as an alternative for Alzheimer's disease drug discovery. *Alzheimers. Dement.* 12, 678–686 (2016).
59. Risner, M. E. *et al.* Efficacy of rosiglitazone in a genetically defined population with mild-to-moderate Alzheimer's disease. *Pharmacogenomics J.* 6, 246–254 (2006).
60. Escribano, L. *et al.* Rosiglitazone rescues memory impairment in Alzheimer's transgenic mice: mechanisms involving a reduced amyloid and tau pathology. *Neuropsychopharmacology* 35, 1593–1604 (2010).
61. Kume, K. *et al.* Effects of telmisartan on cognition and regional cerebral blood flow in hypertensive patients with Alzheimer's disease: Telmisartan on cognition and rCBF in AD. *Geriatr. Gerontol. Int.* 12, 207–214 (2012).
62. Singh, B., Sharma, B., Jaggi, A. S. & Singh, N. Attenuating effect of lisinopril and telmisartan in intracerebroventricular streptozotocin induced experimental dementia of Alzheimer's disease type: possible involvement of PPAR-γ agonistic property. *J. Renin Angiotensin Aldosterone Syst.* 14, 124–136 (2013).
63. Zhou, B. *et al.* Association between exenatide use and incidence of Alzheimer's disease. *Alzheimers Dement. (N. Y.)* 7, e12139 (2021).
64. An, J. *et al.* Exenatide alleviates mitochondrial dysfunction and cognitive impairment in the 5×FAD mouse model of Alzheimer's disease. *Behav. Brain Res.* 370, 111932 (2019).

65. Butchart, J. *et al.* Etanercept in Alzheimer disease: A randomized, placebo-controlled, double-blind, phase 2 trial. *Neurology* 84, 2161–2168 (2015).
66. Torres-Acosta, N., O'Keefe, J. H., O'Keefe, E. L., Isaacson, R. & Small, G. Therapeutic potential of TNF-α inhibition for Alzheimer's disease prevention. *J. Alzheimers. Dis.* 78, 619–626 (2020).
67. Cummings, J. L. *et al.* Effect of dextromethorphan-quinidine on agitation in patients with Alzheimer disease dementia: A randomized clinical trial: A randomized clinical trial. *JAMA* 314, 1242–1254 (2015).
68. Ballard, C., Sharp, S. & Corbett, A. Dextromethorphan and quinidine for treating agitation in patients with Alzheimer disease dementia. *JAMA* 314, 1233–1235 (2015).
69. Zhang, X.-Z., Li, X.-J. & Zhang, H.-Y. Valproic acid as a promising agent to combat Alzheimer's disease. *Brain Res. Bull.* 81, 3–6 (2010).
70. Xuan, A.-G. *et al.* Valproic acid alleviates memory deficits and attenuates amyloid-β deposition in transgenic mouse model of Alzheimer's disease. *Mol. Neurobiol.* 51, 300–312 (2015).
71. Wang, J. *et al.* Valsartan lowers brain beta-amyloid protein levels and improves spatial learning in a mouse model of Alzheimer disease. *J. Clin. Invest.* 117, 3393–3402 (2007).
72. Yang, W.-N. *et al.* The effects of valsartan on cognitive deficits induced by aluminum trichloride and d-galactose in mice. *Neurol. Res.* 36, 651–658 (2014).
73. Fremont, R. *et al.* Tolcapone treatment for cognitive and behavioral symptoms in behavioral variant frontotemporal dementia: A placebo-controlled crossover study. *J. Alzheimers. Dis.* 75, 1391–1403 (2020).
74. Grossmann, K. Anticoagulants for treatment of Alzheimer's disease. *J. Alzheimers. Dis.* 77, 1373–1382 (2020).
75. Wang, J. *et al.* Investigation of nebivolol as a novel therapeutic agent for the treatment of Alzheimer's disease. *J. Alzheimers. Dis.* 33, 1147–1156 (2013).
76. Sanders, O. Sildenafil for the treatment of Alzheimer's disease: A systematic review. *J. Alzheimers Dis. Rep.* 4, 91–106 (2020).
77. Chumakov, I. *et al.* Combining two repurposed drugs as a promising approach for Alzheimer's disease therapy. *Sci. Rep.* 5, 7608 (2015).
78. in t' Veld, B. A. *et al.* Nonsteroidal antiinflammatory drugs and the risk of Alzheimer's disease. *N. Engl. J. Med.* 345, 1515–1521 (2001).
79. Aisen, P. S. Inflammation and Alzheimer's disease: mechanisms and therapeutic strategies. *Gerontology* 43, 143–149 (1997).
80. Cao, S., Yu, L., Mao, J., Wang, Q. & Ruan, J. Uncovering the molecular mechanism of actions between pharmaceuticals and proteins on the AD network. *PLoS One* 10, e0144387 (2015).
81. O'Neill, E., Kwok, B., Day, J. S., Connor, T. J. & Harkin, A. Amitriptyline protects against TNF-α-induced atrophy and reduction in synaptic markers via a Trk-dependent mechanism. *Pharmacol. Res. Perspect.* 4, e00195 (2016).

82. Yang, L., Liu, Y., Wang, Y., Li, J. & Liu, N. Azeliragon ameliorates Alzheimer's disease via the Janus tyrosine kinase and signal transducer and activator of transcription signaling pathway. *Clinics (Sao Paulo)* 76, e2348 (2021).
83. Pilipenko, V. *et al.* Very low doses of muscimol and baclofen ameliorate cognitive deficits and regulate protein expression in the brain of a rat model of streptozocin-induced Alzheimer's disease. *Eur. J. Pharmacol.* 818, 381–399 (2018).
84. Watt, G. & Karl, T. In vivo evidence for therapeutic properties of cannabidiol (CBD) for Alzheimer's disease. *Front. Pharmacol.* 8, 20 (2017).
85. Breitner, J. C. *et al.* Delayed onset of Alzheimer's disease with nonsteroidal anti-inflammatory and histamine H2 blocking drugs. *Neurobiol. Aging* 16, 523–530 (1995).
86. Li, Z.-Y. *et al.* Clemastine attenuates AD-like pathology in an AD model mouse via enhancing mTOR-mediated autophagy. *Exp. Neurol.* 342, 113742 (2021).
87. Beeri, M. S. *et al.* Corticosteroids, but not NSAIDs, are associated with less Alzheimer neuropathology. *Neurobiol. Aging* 33, 1258–1264 (2012).
88. Moura, F. C. D. *et al.* Behavioral, neurochemical and histological changes in the use of low doses of Naltrexone and Donepezil in the treatment in experimental model of Alzheimer's disease by induction of β-Amyloid1-42 in rats. *World Sci. Res.* 6, 5–13 (2019).
89. Chen, S., Liu, A.-R., An, F.-M., Yao, W.-B. & Gao, X.-D. Amelioration of neurodegenerative changes in cellular and rat models of diabetes-related Alzheimer's disease by exendin-4. *Age (Dordr.)* 34, 1211–1224 (2012).
90. Irwin, R. W., Solinsky, C. M. & Brinton, R. D. Frontiers in therapeutic development of allopregnanolone for Alzheimer's disease and other neurological disorders. *Front. Cell. Neurosci.* 8, 203 (2014).
91. Aisen, P. S., Marin, D. B., Brickman, A. M., Santoro, J. & Fusco, M. Pilot tolerability studies of hydroxychloroquine and colchicine in Alzheimer disease. *Alzheimer Dis. Assoc. Disord.* 15, 96–101 (2001).
92. Wilkinson, B. L. *et al.* Ibuprofen attenuates oxidative damage through NOX2 inhibition in Alzheimer's disease. *Neurobiol. Aging* 33, 197.e21–32 (2012).
93. Freiherr, J. *et al.* Intranasal insulin as a treatment for Alzheimer's disease: a review of basic research and clinical evidence. *CNS Drugs* 27, 505–514 (2013).
