# Supplementary material for: Leveraging generative AI to prioritize drug repurposing candidates for Alzheimer’s disease with real-world clinical validation
Source: NPJ Digit Med. 2024 Feb 26;7:46. doi: 10.1038/s41746-024-01038-3 (PMC10897392; doi:10.1038/s41746-024-01038-3)
Supplement: Supplementary file 2 — Reporting Summary [file 41746_2024_1038_MOESM2_ESM.pdf]

nature portfolio

Corresponding author(s): Wei-Qi Wei

Last updated by author(s): 2/2/2024

Reporting Summary

Nature Portfolio wishes to improve the reproducibility of the work that we publish. This form provides structure for consistency and transparency in reporting. For further information on Nature Portfolio policies, see our Editorial Policies and the Editorial Policy Checklist.

Statistics

For all statistical analyses, confirm that the following items are present in the figure legend, table legend, main text, or Methods section.

n/a	Confirmed	
☐	☒	The exact sample size (n) for each experimental group/condition, given as a discrete number and unit of measurement
☐	☒	A statement on whether measurements were taken from distinct samples or whether the same sample was measured repeatedly
☐	☒	The statistical test(s) used AND whether they are one- or two-sided<br>*Only common tests should be described solely by name; describe more complex techniques in the Methods section.*
☐	☒	A description of all covariates tested
☐	☒	A description of any assumptions or corrections, such as tests of normality and adjustment for multiple comparisons
☐	☒	A full description of the statistical parameters including central tendency (e.g. means) or other basic estimates (e.g. regression coefficient) AND variation (e.g. standard deviation) or associated estimates of uncertainty (e.g. confidence intervals)
☐	☒	For null hypothesis testing, the test statistic (e.g. F, t, r) with confidence intervals, effect sizes, degrees of freedom and P value noted<br>*Give P values as exact values whenever suitable.*
☒	☐	For Bayesian analysis, information on the choice of priors and Markov chain Monte Carlo settings
☒	☐	For hierarchical and complex designs, identification of the appropriate level for tests and full reporting of outcomes
☐	☒	Estimates of effect sizes (e.g. Cohen's d, Pearson's r), indicating how they were calculated

Our web collection on statistics for biologists contains articles on many of the points above.

Software and code

Policy information about availability of computer code

Data collection	We extracted EHR data from the databases used in this study using SQL queries.
Data analysis	Propensity score matching was performed using the MatchIt R package. All survival analyses were performed using Cox proportional hazards regression models. Meta-analysis of hazard ratios was performed using NCSS statistical software.<br><br>The source code associated with this study is publicly available at: https://github.com/monikagrabowska/GPT4_AD_Drug_Repurposing.

For manuscripts utilizing custom algorithms or software that are central to the research but not yet described in published literature, software must be made available to editors and reviewers. We strongly encourage code deposition in a community repository (e.g. GitHub). See the Nature Portfolio guidelines for submitting code & software for further information.

Data

Policy information about availability of data

All manuscripts must include a data availability statement. This statement should provide the following information, where applicable:

- Accession codes, unique identifiers, or web links for publicly available datasets
- A description of any restrictions on data availability
- For clinical datasets or third party data, please ensure that the statement adheres to our policy

The VUMC dataset used in this study is available upon request from the corresponding authors and subsequent institutional approval. The All of Us dataset can be accessed through the Researcher Workbench by following the detailed data application process outlined at https://www.researchallofus.org.

Research involving human participants, their data, or biological material

Policy information about studies with human participants or human data. See also policy information about sex, gender (identity/presentation), and sexual orientation and race, ethnicity and racism.

Reporting on sex and gender	We applied 2:1 propensity score matching with sex, race, length of EHR after age 65, and relevant drug-specific comorbidities as covariates to form comparable drug-exposed and unexposed cohorts for each suggested drug repurposing candidate. We do not share any individual-level data.
Reporting on race, ethnicity, or other socially relevant groupings	We applied 2:1 propensity score matching with sex, race, length of EHR after age 65, and relevant drug-specific comorbidities as covariates to form comparable drug-exposed and unexposed cohorts for each suggested drug repurposing candidate. We do not share any individual-level data.
Population characteristics	Sex, race, length of EHR after age 65, relevant drug-specific comorbidities, AD diagnosis, time to AD diagnosis, drug exposure
Recruitment	N/A (retrospective study using de-identified EHR data)
Ethics oversight	N/A (not human subjects research)

Note that full information on the approval of the study protocol must also be provided in the manuscript.

Field-specific reporting

Please select the one below that is the best fit for your research. If you are not sure, read the appropriate sections before making your selection.

☒ Life sciences ☐ Behavioural & social sciences ☐ Ecological, evolutionary & environmental sciences

For a reference copy of the document with all sections, see nature.com/documents/nr-reporting-summary-flat.pdf

Life sciences study design

All studies must disclose on these points even when the disclosure is negative.

Sample size	No sample size calculation, leveraging all available EHR data
Data exclusions	We excluded individuals with a diagnosis of non-Alzheimer's dementia (vascular dementia, diffuse Lewy body disease, frontotemporal dementia, mixed dementia, and dementia associated with Parkinson's disease), individuals without EHR follow-up after age 65, and individuals with missing demographic characteristics.
Replication	Analysis performed in 2 independent EHR databases, with meta-analysis
Randomization	Propensity score matching
Blinding	Blinding not relevant (observational study using EHR databases)

Reporting for specific materials, systems and methods

We require information from authors about some types of materials, experimental systems and methods used in many studies. Here, indicate whether each material, system or method listed is relevant to your study. If you are not sure if a list item applies to your research, read the appropriate section before selecting a response.

Materials & experimental systems

n/a	Involved in the study	
☒	☐	Antibodies
☒	☐	Eukaryotic cell lines
☒	☐	Palaeontology and archaeology
☒	☐	Animals and other organisms
☒	☐	Clinical data
☒	☐	Dual use research of concern
☒	☐	Plants

Methods

n/a	Involved in the study	
☒	☐	ChIP-seq
☒	☐	Flow cytometry
☒	☐	MRI-based neuroimaging

Plants

Seed stocks

Report on the source of all seed stocks or other plant material used. If applicable, state the seed stock centre and catalogue number. If plant specimens were collected from the field, describe the collection location, date and sampling procedures.

Novel plant genotypes

Describe the methods by which all novel plant genotypes were produced. This includes those generated by transgenic approaches, gene editing, chemical/radiation-based mutagenesis and hybridization. For transgenic lines, describe the transformation method, the number of independent lines analyzed and the generation upon which experiments were performed. For gene-edited lines, describe the editor used, the endogenous sequence targeted for editing, the targeting guide RNA sequence (if applicable) and how the editor was applied.

Authentication

Describe any authentication procedures for each seed stock used or novel genotype generated. Describe any experiments used to assess the effect of a mutation and, where applicable, how potential secondary effects (e.g. second site T-DNA insertions, mosiacism, off-target gene editing) were examined.
